# Association of Life’s Essential 8 with the prevalence and mortality of chronic obstructive pulmonary disease

**DOI:** 10.3389/fmed.2025.1530493

**Published:** 2025-04-25

**Authors:** Yushan Shi, Di Huang, Yaobei Liu, Ning Huang

**Affiliations:** ^1^Department of Laboratory, Affiliated Hospital of Shandong University of Traditional Chinese Medicine, Jinan, Shandong, China; ^2^Shandong University of Traditional Chinese Medicine, Jinan, Shandong, China; ^3^Department of Respiratory and Critical Care Medicine, The Affiliated Hospital of Shandong University of Traditional Chinese Medicine, Jinan, China

**Keywords:** Life’s Essential 8, all-cause mortality, CVD mortality, COPD, NHANES

## Abstract

**Objective:**

To study the correlation between Life’s Essential 8 (LE8) and the occurrence of chronic obstructive pulmonary disease (COPD) among US adults, as well as the association between LE8 and all-cause and cardiovascular disease (CVD) mortality among individuals with COPD.

**Methods:**

Data from National Health and Nutrition Examination Survey (2005–2018 year) were analyzed. The correlation between LE8 scores and the prevalence of COPD was evaluated using logistic regression models. Additionally, the Cox proportional hazards model was applied to investigate how LE8 scores relate to the risk of mortality from all causes and cardiovascular diseases. To ensure the robustness of the findings, sensitivity analyses and subgroup analyses were performed.

**Results:**

In the overall population, an inverse relationship was observed between a 10-point increase in LE8 score and the risk of COPD [OR = 0.78, 95%CI (0.75 ~ 0.82), *p* < 0.001]. Those diagnosed with COPD experienced a 65% increased rate of all-cause mortality and 5% higher rate of mortality due to cardiovascular diseases compared to the non-COPD group. Within the COPD patient cohort, an inverse relationship was similarly observed between a 10-point increase in the LE8 score and the risk of all-cause mortality [HR = 0.87, 95%CI (0.8 ~ 0.95), *p* = 0.002]. However, no significant association was found between the LE8 score and CVD mortality [HR = 0.83, 95%CI (0.68 ~ 1.02), *p* = 0.073]. In further exploration through subgroup analysis, no statistically significant interactions were found, suggesting consistency across different demographic or clinical subgroups.

**Conclusion:**

Higher LE8 adherence is linked to lower COPD prevalence and all-cause mortality, yet no clear link to CVD mortality was found. This highlights the need for more extensive research to clarify LE8’s role in CVD outcomes.

## Introduction

Chronic obstructive pulmonary disease (COPD) is a prevalent respiratory condition marked by ongoing respiratory issues and a gradual decline in airflow (1). COPD ranks as the foremost cause of disability and the third leading cause of mortality in the United States, this prevalent condition not only diminishes the quality of life for those affected but also imposes a considerable economic strain on the healthcare system, underscoring the imperative for enhanced clinical interventions and preventive measures (2). Between 1990 and 2017, there was a notable increase in the global prevalence of COPD, rising by 5.9% (3). The World Health Organization (WHO) has projected that by the year 2040, the annual death toll attributed to COPD could soar to 4.4 million individuals (4, 5). The natural course and prognosis of COPD are influenced by various clinical factors, about 30% of COPD patients die from heart-related problems (6). COPD patients are at an increased risk of cardiovascular disease (CVD), which exacerbates their prognosis (7). COPD and CVD share several common risk factors, including smoking and a sedentary lifestyle (8). Therefore, identifying modifiable risk factors is crucial for preventing COPD complications and reducing mortality.

The American Heart Association’s “Life’s Essential 8” (LE8) score, an update to the initial cardiovascular health (CVH) metrics, has been linked to a reduced risk of chronic disease (9–11), and all-cause mortality (12). A nonlinear dose–response relationship was found between LE8 and COPD in adults (13). However, its relationship with long-term mortality in COPD patients aged 40 years and above remains understudied.

This study seeks to address this gap by examining the connection between the updated LE8 metrics and the risk of all-cause and CVD mortality among COPD patients in the United States. The overarching goal is to identify a holistic prevention strategy that could potentially enhance survival rates and improve outcomes for the COPD patient population.

## Methods

### Data sources

The National Health and Nutrition Examination Survey (NHANES), led by the National Center for Health Statistics (NCHS), uses a complex sampling method to gather data from a representative sample of the US civilian population. The survey collects detailed information on demographics, diet, socioeconomic status, and health, along with blood samples at mobile centers. Its standardized protocols for data collection ensure high reproducibility and comparability across studies. All NHANES data are public, and the methodology is detailed in their guidelines. The study was approved by the NCHS ethics board, and all participants gave informed consent. As a publicly available, de-identified dataset, no additional ethical approval or authorization was required for this secondary analysis.

This research follows the Strengthening the Reporting of Observational Studies in Epidemiology (STROBE) reporting guidelines for observational studies to ensure clear and rigorous reporting (14).

### Study design and population

**Figure 1:**
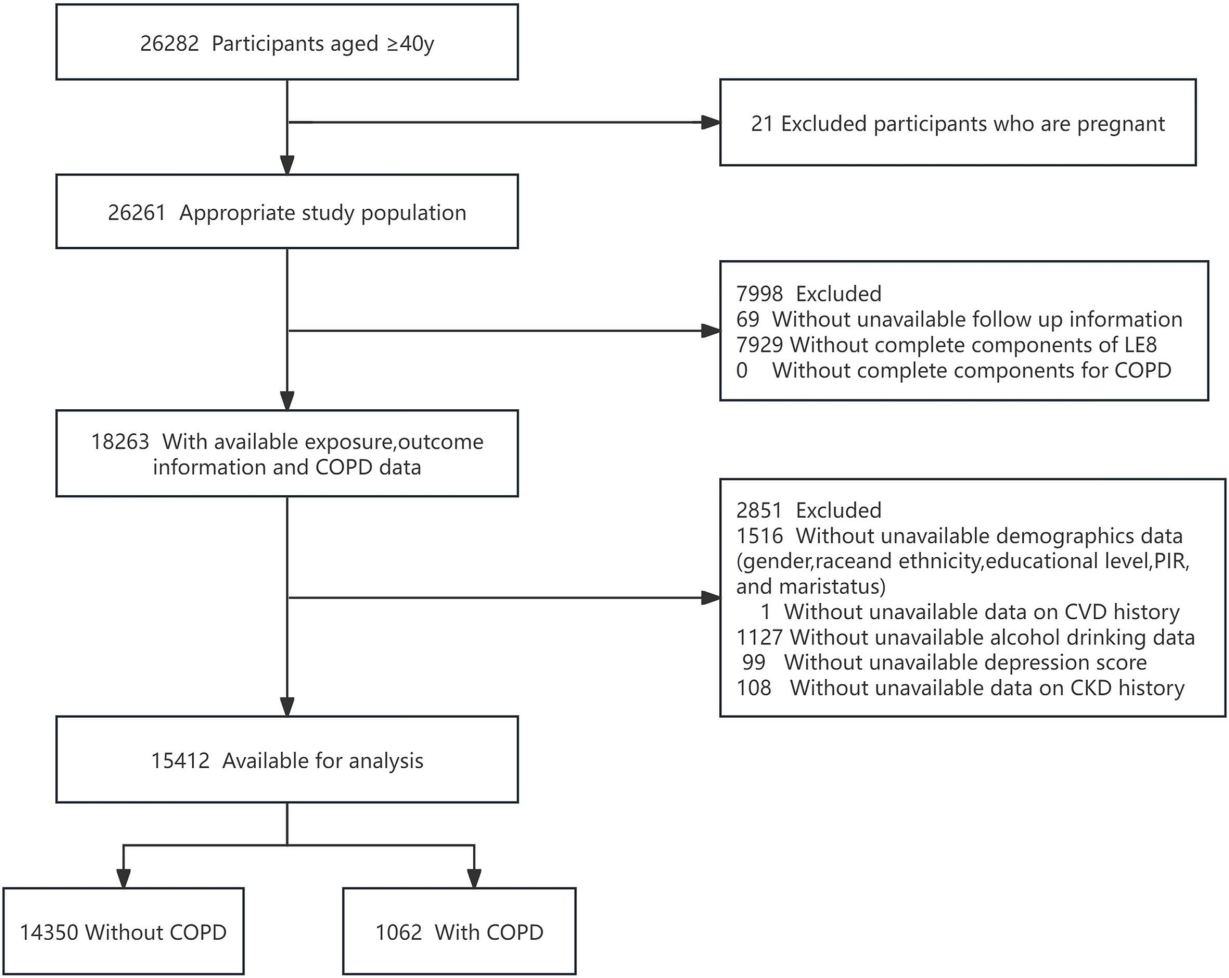
Flow diagram of the screening and enrollment of study participants. COPD, chronic obstructive pulmonary disease; PIR, poverty income ratio; CVD, cardiovascular disease; CKD, chronic kidney disease. Data are from the NHANES (2005–2018)-linked mortality file.

The study analyzed data from seven NHANES cycles spanning 2005–2018, initially considering 26,282 participants aged 40 and above. Exclusions were made for pregnant participants (21), those missing follow-up data (69), and data on LE8 components (7,929), demographics (1,516), CVD history (1), alcohol consumption (1,127), depression scores (99), and CKD history (108). After applying these criteria, the study included 14,350 individuals without COPD and 1,062 with COPD (shown in Figure 1).

### Definitions of LE8

The LE8 score is determined by eight key health indicators: four behaviors—diet, physical activity, nicotine exposure, and sleep—and four health factors, including body mass index, blood lipids, blood glucose, and blood pressure. The specific calculations for these LE8 metrics, tailored to NHANES data, have been published and are available in Supplementary Table S1 (15, 16). The dietary aspect of the study was assessed utilizing the criteria set by the Healthy Eating Index (HEI) 2015 (17). Comprehensive details regarding the HEI-2015’s components and its scoring guidelines are provided in Supplementary Table S2.

Physical activity (PA) was measured by self-reported weekly time spent in moderate to vigorous exercise. Nicotine exposure was evaluated through self-reported smoking and use of nicotine delivery systems, considering both personal use and passive exposure from household smokers. Sleep health was determined by self-reported nightly sleep duration. During physical exams, blood pressure, height, and weight were directly measured, with Body Mass Index (BMI) calculated as weight in kilograms divided by the square of height in meters. Blood samples were analyzed in central labs for blood lipids, plasma glucose, and hemoglobin A1c levels. Self-reported use of medications for hypercholesterolemia, hypertension, and diabetes was also recorded.

The eight CVH metrics, each scored from 0 to 100, were averaged to determine the overall LE8 score. The aggregated CVH score categorized individuals into high (80–100), moderate (50–79), and low (0–49) cardiovascular health levels (15).

### Definition of COPD

Referring to previous studies (18–20), it can be diagnosed as COPD by fulfilling any of the following criteria: (1) A ratio of Forced Expiratory Volume in one second (FEV1) to Forced Vital Capacity (FVC) of less than 0.7 after the use of bronchodilators; (2) Positive responses to questions from the MCQ questionnaires “mcq160g” or “mcq160p” regarding a previous diagnosis of emphysema; (3) Being over 40 years old, having a history of smoking or chronic bronchitis, and long-term use of medications such as selective phosphodiesterase-4 inhibitors, mast cell stabilizers, leukotriene modifiers, and inhaled corticosteroids.

### Assessment of outcomes

Adult participants’ mortality data from NHANES were sourced until December 31, 2019, through the National Death Index (NDI). The follow-up period ranged from the interview date to the date of death for deceased participants or until the end of 2019 for those still living. We used the International Classification of Diseases, 10th Edition (ICD-10), to identify all-cause mortality, encompassing any reason for death, and CVD mortality, categorized under heart diseases with codes I00–I09, I11, I13, and I20–I51 (21).

### Covariates

Potential covariates in this study were identified through questionnaires and measurements. Age was divided into three groups: 40–49, 50–59, and 60 years or older. Race/ethnicity categories included Mexican American, non-Hispanic black, non-Hispanic white, other Hispanic, and others. Marital status encompassed married, never married, living with a partner, and other statuses such as widowed, divorced, or separated. Education levels were categorized as less than high school, high school or equivalent, and above high school. The poverty income ratio (PIR) was divided into low income (below 1.3), middle income (1.3–3.5), and high income (above 3.5). Self-reported CVD history included conditions such as heart failure, coronary heart disease, angina, heart attack, or stroke (22). The Chronic kidney disease (CKD) was defined as an estimated glomerular filtration rate (eGFR) < 60 mL/min per 1.73m^2^ or albuminuria ≥30 mg/g, or both (23). The eGFR was determined using the Chronic Kidney Disease Epidemiology Collaboration (CKD-EPI) formula based on serum creatinine levels (24). And albuminuria was calculated as the ratio of urine albumin to creatinine. Drinking status was self-reported and categorized as never, former, mild, moderate, or heavy (16). Mental health was measured using the Patient Health Questionnaire 9 (PHQ-9); participants who scored ≥10 was considered to have depression (25).

### Statistical analysis

Participant characteristics at the start of the study were stratified by levels of CVH. For continuous variables with a normal distribution, we reported the mean values along with their standard deviations. Skewed continuous variables were described using medians and interquartile ranges. Categorical variables were depicted through counts and frequencies presented as percentages. The Chi-square test was employed to identify significant differences in categorical variables across various levels of CVH, while the Analysis of Variance (ANOVA) was applied to assess variations in continuous variables.

A multivariable logistic regression analysis assessed the link between LE8 scores and the risk of developing COPD. Additionally, a Cox proportional hazards regression analysis was used to study how COPD relates to the risk of all-cause and CVD mortality and to determine the influence of LE8 scores on mortality within the COPD group. The LE8 score was considered both as a continuous variable, with calculations for a 10-point increase represented by odds ratios (OR) or hazard ratios (HR), and as a categorical variable with classifications of low, moderate, and high CVH. The analysis proceeded through three models: Model 1 served as the basic model without any adjustments. Model 2 included adjustments for age, sex, race/ethnicity, and marital status. Model 3 expanded the adjustments to encompass educational level, poverty income ratio (PIR), history of CVD and CKD, drinking habits, and depression status.

To identify possible interactions, we conducted stratified analyses based on several factors: age groups (below 65 years and 65 years and older), gender (male and female), PIR categories (up to 1.3, between 1.3 and 3.5, and above 3.5), and the presence or absence of CVD and CKD history.

To ensure the reliability of our results, we conducted further sensitivity analyses. Initially, we removed participants who died within the first 2 years of the study to reduce the possible influence of reverse causality. Next, we incorporated LE8 scores into the regression models based on quartile distribution and reassessed their correlation with all-cause and cardiovascular disease (CVD) mortality among COPD patients.

R Statistical Software (Version 4.2.2. The R Foundation) and the Free Statistics Analysis Platform (Version 1.9) were applied to all analyses. Statistical significance was set at a *p*-value of less than 0.05 for all tests.

## Results

### Baseline characteristics

The study enrolled 15,412 adults aged 40 and older, among whom 1,062 had COPD, resulting in 337 deaths, including 62 due to CVD. Participants had a mean age of 59.4 years and a mean total CVH score of 63.9 points. Men made up 48.8% of the study population.

Higher CVH scores were associated with being younger, female gender, higher education levels, non-Hispanic white race, higher income, better kidney function (eGFR), and a lower urine albumin-to-creatinine ratio (UACR). Participants with higher CVH scores also tended to consume alcohol in moderation and had no history of CVD, depression, or CKD. Further details are provided in Table 1.

**Table 1:** Baseline characteristics by CVH score level.

Variables	Total (*n* = 15,412)	Low CVH (*n* = 2,479)	Moderate CVH (*n* = 10,810)	High CVH (*n* = 2,123)	*p-*value
Age, years Mean ± SD	59.4 ± 12.1	60.4 ± 11.3	59.8 ± 12.2	56.2 ± 12.1	< 0.001
Age, n (%)					< 0.001
40–49	4,029 (26.1)	516 (20.8)	2,715 (25.1)	798 (37.6)	
50–59	3,762 (24.4)	611 (24.6)	2,620 (24.2)	531 (25)	
≥60	7,621 (49.4)	1,352 (54.5)	5,475 (50.6)	794 (37.4)	
Sex, n (%)					< 0.001
Female	7,895 (51.2)	1,327 (53.5)	5,300 (49)	1,268 (59.7)	
Male	7,517 (48.8)	1,152 (46.5)	5,510 (51)	855 (40.3)	
Race/ethnicity, n (%)					< 0.001
Mexican American	2047 (13.3)	319 (12.9)	1,517 (14)	211 (9.9)	
Non-Hispanic Black	3,106 (20.2)	706 (28.5)	2,180 (20.2)	220 (10.4)	
Non-Hispanic White	7,691 (49.9)	1,129 (45.5)	5,353 (49.5)	1,209 (56.9)	
Other Hispanic	1,351 (8.8)	220 (8.9)	956 (8.8)	175 (8.2)	
Others	1,217 (7.9)	105 (4.2)	804 (7.4)	308 (14.5)	
Marital status, n (%)					< 0.001
Living with partner	690 (4.5)	141 (5.7)	475 (4.4)	74 (3.5)	
Married	9,096 (59.0)	1,210 (48.8)	6,409 (59.3)	1,477 (69.6)	
Never married	1,180 (7.7)	225 (9.1)	812 (7.5)	143 (6.7)	
Others	4,446 (28.8)	903 (36.4)	3,114 (28.8)	429 (20.2)	
PIR, n (%)					< 0.001
<1.3	4,067 (26.4)	999 (40.3)	2,773 (25.7)	295 (13.9)	
1.3–3.5	5,885 (38.2)	1,023 (41.3)	4,227 (39.1)	635 (29.9)	
>3.5	5,460 (35.4)	457 (18.4)	3,810 (35.2)	1,193 (56.2)	
Education level, n (%)					< 0.001
Less than high school	3,584 (23.3)	857 (34.6)	2,485 (23)	242 (11.4)	
High school or equivalent	3,606 (23.4)	677 (27.3)	2,646 (24.5)	283 (13.3)	
Above high school	8,222 (53.3)	945 (38.1)	5,679 (52.5)	1,598 (75.3)	
Drinking status, n (%)					< 0.001
Never	2066 (13.4)	296 (11.9)	1,471 (13.6)	299 (14.1)	
Former	3,176 (20.6)	735 (29.6)	2,199 (20.3)	242 (11.4)	
Mild	5,828 (37.8)	724 (29.2)	4,054 (37.5)	1,050 (49.5)	
Moderate	2,195 (14.2)	314 (12.7)	1,522 (14.1)	359 (16.9)	
Heavy	2,147 (13.9)	410 (16.5)	1,564 (14.5)	173 (8.1)	
Depression, n (%)					< 0.001
No	14,092 (91.4)	2045 (82.5)	9,994 (92.5)	2053 (96.7)	
Yes	1,320 (8.6)	434 (17.5)	816 (7.5)	70 (3.3)	
CVD, n (%)					< 0.001
No	13,063 (84.8)	1819 (73.4)	9,257 (85.6)	1987 (93.6)	
Yes	2,349 (15.2)	660 (26.6)	1,553 (14.4)	136 (6.4)	
CKD, n (%)					< 0.001
No	11,900 (77.2)	1,542 (62.2)	8,456 (78.2)	1902 (89.6)	
Yes	3,512 (22.8)	937 (37.8)	2,354 (21.8)	221 (10.4)	
COPD, n (%)					< 0.001
No	14,350 (93.1)	2,167 (87.4)	10,118 (93.6)	2065 (97.3)	
Yes	1,062 (6.9)	312 (12.6)	692 (6.4)	58 (2.7)	
eGFR, mL/(min·1.73 m^2^)	84.3 ± 20.8	81.2 ± 23.7	84.2 ± 20.6	88.4 ± 17.3	< 0.001
UACR, mean (SE), mg/g	7.8 (4.9, 15.8)	11.3 (6.2, 34.9)	7.6 (4.8, 15.0)	6.4 (4.4, 10.4)	< 0.001
Total CVH score	63.9 ± 14.1	41.9 ± 6.3	64.6 ± 8.1	85.8 ± 4.7	< 0.001
Diet score, median (IQR)	50.0 (25.0, 80.0)	25.0 (0.0, 50.0)	50.0 (25.0, 80.0)	80.0 (50.0, 80.0)	< 0.001
PA score, median (IQR)	100.0 (0.0, 100.0)	0.0 (0.0, 60.0)	100.0 (0.0, 100.0)	100.0 (100.0, 100.0)	< 0.001
Nicotine exposure score, median (IQR)	80.0 (75.0, 100.0)	55.0 (0.0, 100.0)	80.0 (75.0, 100.0)	100.0 (80.0, 100.0)	< 0.001
Sleep health score, Mean ± SD	81.1 ± 25.7	65.4 ± 31.2	82.4 ± 24.3	92.9 ± 14.5	< 0.001
BMI score, median (IQR)	70.0 (30.0, 70.0)	30.0 (15.0, 70.0)	70.0 (30.0, 70.0)	100.0 (70.0, 100.0)	< 0.001
Blood lipids score, median (IQR)	60.0 (40.0, 80.0)	40.0 (20.0, 60.0)	60.0 (40.0, 80.0)	80.0 (60.0, 100.0)	< 0.001
Blood glucose score, mean ± SD	76.6 ± 28.2	55.0 ± 29.3	78.0 ± 26.9	95.0 ± 14.2	< 0.001
BP score, median (IQR)	55.0 (30.0, 80.0)	30.0 (5.0, 55.0)	55.0 (30.0, 80.0)	100.0 (75.0, 100.0)	< 0.001

COPD, chronic obstructive pulmonary disease; CKD, chronic kidney disease; CVD, cardiovascular disease; CVH, cardiovascular health score; PIR, poverty income ratio. CVH scores range from 0 to 100 and are classified into low CVH (0–49), moderate CVH (50–79), and high CVH (80–100).

### Association of LE8 scores with the risk of COPD

Table 2 displays the rates of COPD in relation to LE8 scores and the correlation between these scores and the prevalence of COPD. Each increment of 10 points in the LE8 score correlated with a 22% reduced likelihood of COPD in the adjusted model (Model 3), with an odds ratio (OR) of 0.78 and a 95% confidence interval (CI) ranging from 0.75 to 0.82. When categorized, both moderate and high levels of CVH showed a lower prevalence of COPD compared to low CVH levels, with moderate CVH having an OR of 0.56 (95% CI: 0.48–0.65) and high CVH having an OR of 0.28 (95% CI: 0.21–0.38).

**Table 2:** Association between Life’s Essential 8 score and the risk of COPD.

Variable	Cases/participants (%)	Model 1	*p-*value	Model 2	*p*-value	Model 3	*p*-value
		OR (95%CI)		OR (95%CI)		OR (95%CI)	
Per 10 score increase	1062/15412 (6.9)	0.73 (0.7 ~ 0.76)	<0.001	0.72 (0.69 ~ 0.75)	<0.001	0.78 (0.75 ~ 0.82)	<0.001
Subgroups							
Low CVH	312/2479 (12.6)	1(Ref)		1(Ref)		1(Ref)	
Moderate CVH	692/10810 (6.4)	0.48 (0.41 ~ 0.55)	<0.001	0.46 (0.39 ~ 0.53)	<0.001	0.56 (0.48 ~ 0.65)	<0.001
High CVH	58/2123 (2.7)	0.2 (0.15 ~ 0.26)	<0.001	0.2 (0.15 ~ 0.26)	<0.001	0.28 (0.21 ~ 0.38)	<0.001
*P* for Trend			<0.001		<0.001		<0.001

CVH, cardiovascular health; OR, odd ratio; CI, confidence interval. Model 1 was not adjusted. Model 2 was adjusted for age, sex, race/ethnicity, and marital status. Model 3 was adjusted for age, sex, race/ethnicity, marital status, educational level, PIR, CVD history, CKD history, drinking status, and depression.

### Association of LE8 scores with all-cause and CVD mortality

Table 3 details mortality rates for individuals with COPD, contrasting all-cause and CVD mortality with those without the condition and examining the impact of different CVH levels on these rates. Across the entire study group, COPD was associated with significantly higher all-cause mortality rates (HR, 1.65; 95% CI, 1.47–1.86) in the adjusted analysis, but this did not translate to an increased risk for CVD mortality (HR, 1.05; 95% CI, 0.80–1.37).

**Table 3:** Association of Life’s Essential 8 score with all-cause, and CVD mortality.

Variable	Cases/participants (%)	Model 1	*p*-value	Model 2	*p*-value	Model 3	*p*-value
		HR (95%CI)		HR (95%CI)		HR (95%CI)	
All-cause mortality							
Total population							
Without COPD	1936/14350 (13.5)	1(Ref)		1(Ref)		1(Ref)	
With COPD	337/1062 (31.7)	2.47 (2.2 ~ 2.77)	<0.001	1.78 (1.58 ~ 2)	<0.001	1.65 (1.47 ~ 1.86)	<0.001
Participants with COPD							
Per 10 score increase	337/2062 (31.7)	0.85 (0.79 ~ 0.92)	<0.001	0.79 (0.73 ~ 0.86)	<0.001	0.87 (0.8 ~ 0.95)	0.002
Subgroups							
Low CVH	115/312 (36.9)	1(Ref)		1(Ref)		1(Ref)	
Moderate CVH	211/629 (30.5)	0.73 (0.59 ~ 0.92)	0.008	0.65 (0.52 ~ 0.82)	<0.001	0.8 (0.63 ~ 1.01)	0.061
High CVH	11/58 (19)	0.4 (0.22 ~ 0.75)	0.004	0.26 (0.14 ~ 0.49)	<0.001	0.44 (0.23 ~ 0.83)	0.011
*P* for trend			<0.001		<0.001		0.006
CVD mortality							
Total population							
Without COPD	530/14350 (3.7)	1(Ref)		1(Ref)		1(Ref)	
With COPD	62/1062 (5.8)	1.66 (1.27 ~ 2.15)	<0.001	1.16 (0.89 ~ 1.52)	0.261	1.05 (0.8 ~ 1.37)	0.722
Participants with COPD							
Per 10 score increase	62/1062 (5.8)	0.82 (0.69 ~ 0.97)	0.024	0.76 (0.63 ~ 0.92)	0.005	0.83 (0.68 ~ 1.02)	0.073
Subgroups							
Low CVH	23/312 (7.4)	1(Ref)		1(Ref)		1(Ref)	
Moderate CVH	36/692 (5.2)	0.64 (0.38 ~ 1.07)	0.091	0.58 (0.34 ~ 0.98)	0.042	0.68 (0.39 ~ 1.18)	0.171
High CVH	3/58 (5.2)	0.57 (0.17 ~ 1.91)	0.366	0.39 (0.11 ~ 1.31)	0.128	0.57 (0.16 ~ 2.02)	0.38
*P* for trend			0.099		0.026		0.161

COPD, chronic obstructive pulmonary disease; CVH, cardiovascular health; CVD, cardiovascular disease; HR, hazard ratio; CI, confidence interval. Model 1 was not adjusted. Model 2 was adjusted for age, sex, race/ethnicity, and marital status. Model 3 was adjusted for age, sex, race/ethnicity, marital status, educational level, PIR, CVD history, CKD history, drinking status, and depression.

Among COPD patients, a 10-point increase in the LE8 score corresponded to a 13% decrease in all-cause mortality risk (HR, 0.87; 95% CI, 0.80–0.95). Moreover, higher CVH levels were linked to reduced all-cause mortality, with moderate and high CVH levels showing respective hazard ratios of 0.80 (95% CI, 0.63–1.01) and 0.44 (95% CI, 0.23–0.83) when compared to the low CVH group. Notably, no clear pattern of increased CVD mortality risk was associated with varying levels of CVH (moderate CVH: HR, 0.68; 95% CI, 0.39–1.18; high CVH: HR, 0.57; 95% CI, 0.16–2.02).

### Stratified and sensitivity analysis

**Figure 2:**
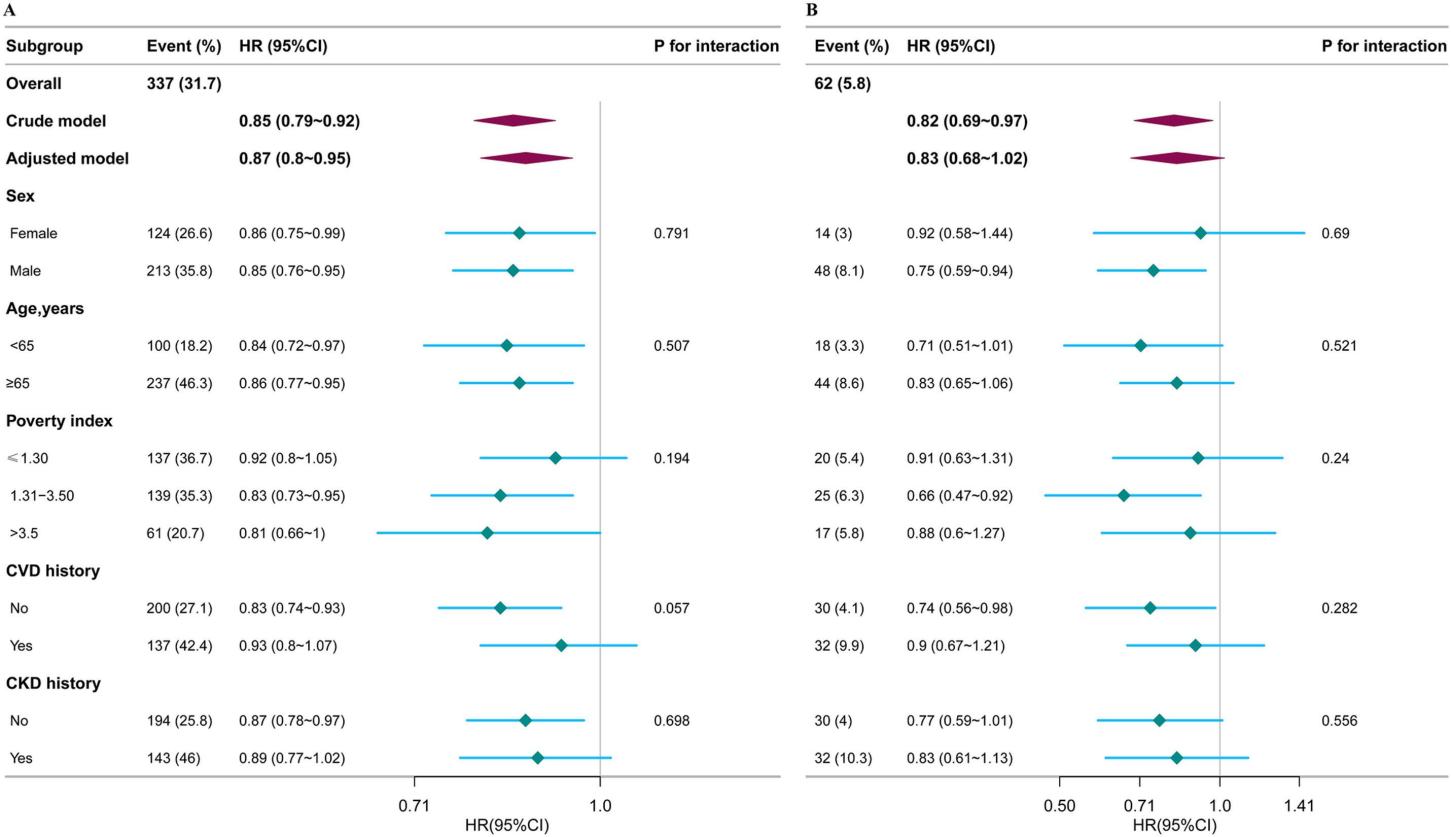
Subgroup analysis of the association between the Life’s Essential 8 scores and mortality from all-causes **(A)** and cardiovascular disease mortality **(B)**. HRs were calculated as per 10 points increase in the LE8 score. Each stratification was adjusted for age, sex, race/ethnicity, marital status, educational level, PIR, CVD history, CKD history, drinking status, and depression. HR, hazard ratio; CI, confidence interval.

Stratified analyses, depicted in Figures 2A,B, showed no significant interaction effects, with all interaction *p*-values exceeding 0.05.

The robustness of our findings was upheld in sensitivity analyses. Excluding participants who died within the first 2 years post-enrollment did not significantly alter the observed reduction in all-cause and CVD mortality risks associated with higher LE8 scores, as evidenced in Supplementary Table S3. Consistent results were found when re-analyzing the data using LE8 score quartiles, as detailed in Supplementary Table S4.

## Discussion

This study utilized data from the National Health and Nutrition Examination Survey (NHANES) spanning 2005–2018 to explore the correlation between LE8 scores and the risk of developing COPD among US adults, as well as the relationship between LE8 scores and all-cause and cardiovascular disease (CVD) mortality among individuals with COPD. The findings revealed an inverse association between LE8 scores and the risk of COPD, as well as a correlation between higher LE8 scores and reduced all-cause mortality risk in COPD patients. However, no significant association was found between LE8 scores and CVD mortality risk. Stratified and sensitivity analyses confirmed the reliability of our findings.

Evidence increasingly suggests that adherence to a healthy lifestyle, including regular physical activity and a high-quality diet, is beneficial for individuals with COPD. Physical activity not only reduces mortality risks and cardiovascular strain but also extends life expectancy (26). Moreover, a nutritious diet is linked to improved lung function and a lower risk of developing COPD (27). Previous studies on COPD have often concentrated on single factors, neglecting a comprehensive approach to patient care. The LE8 score, introduced by the American Heart Association (AHA), offers a holistic assessment that encompasses four health behaviors—diet, physical activity, nicotine exposure, and sleep health—and four health factors—body mass index, blood lipids, blood glucose, and blood pressure. This tool is designed to facilitate the evaluation of ideal health status in clinical settings and to inform rehabilitation strategies for patients with COPD. Our study investigated the correlation between the LE8 score and COPD incidence. Even after adjusting for various covariates, our findings remained significant, indicating that a higher LE8 score is linked to a reduced risk of developing COPD, which underscores the importance of a multifaceted approach to health in mitigating COPD risks.

A cohort study of the Chinese population aged 18–40 years indicates that levels of LE8 have an inverse gradient association with all-cause mortality (28). A negative linear correlation was also discovered among adults in the United States (29, 30). Among U.S. cancer survivors and type 2 diabetes mellitus, there was an inverse relationship between higher LE8 and the reduced risk of death from all causes and CVD (31–33). While in patients with rheumatoid arthritis (RA), the inverse relationship was robust for all-cause mortality but not for CVD mortality (34). Similarly, our study reveals a significant correlation between increased LE8 score and all-cause mortality in COPD, but not with cardiovascular mortality, which we speculate may be related to the relatively small sample size for CVD specific mortality. Unlike the aforementioned study, our research focuses on patients with COPD who are over the age of 40. Our study offers significant guidance for the rehabilitation of COPD patients, uniquely establishing a link between the LE8 score and both COPD prevalence and all-cause mortality rates. The insights gained could be instrumental for the prevention and management of COPD among adults in the United States.

While our findings highlight the protective role of LE8 in a U.S. cohort, cultural and healthcare disparities may influence the generalizability of these associations. For example, smoking prevalence, dietary patterns and healthcare access differ markedly between the U.S. and other regions may modulate the relationship between LE8 metrics and COPD outcomes, underscoring the need for region-specific validations.

The observed inverse association between LE8 scores and COPD outcomes may be attributed to several potential mechanisms. Adherence to the LE8 metrics, which encompass healthy behaviors such as regular physical activity, a balanced diet, and adequate sleep, likely modulates systemic inflammation, a key factor in COPD pathogenesis (35). Moreover, improved cardiopulmonary reserve resulting from better cardiovascular health could enhance overall resilience and reduce mortality risk in COPD patients (36). Future research should further explore these pathways to elucidate the precise mechanisms underlying the observed associations.

This study has several limitations. First, its observational design precludes causal inference, and residual confounding may persist despite multivariable adjustments. Second, self-reported data on behaviors (e.g., physical activity, smoking) are susceptible to recall bias. Third, NHANES lacks detailed COPD severity classifications and exacerbation histories, which may modulate LE8’s associations with outcomes. Our analysis focused on U.S. adults, and cultural, lifestyle, and healthcare system differences may limit direct applicability to other populations. Future studies should validate these findings in additional regions cohorts. Which will address regional disparities in risk factor profiles and healthcare systems. The limited number of cardiovascular disease-specific mortality events resulted in low statistical power, which reduced our ability to detect the association between LE8 scores and cardiovascular disease mortality. Future studies with larger sample sizes or longer follow-up periods are needed to validate these findings. Despite these limitations, our use of a large, nationally representative cohort and rigorous statistical methods provides valuable insights into LE8’s role in COPD prevention.

## Conclusion

The study shows that higher LE8 scores correlate with a lower risk of COPD and decreased all-cause mortality in affected individuals, highlighting the value of improved cardiovascular health in COPD management. Further research is needed to clarify LE8’s impact on COPD outcomes.

## Data Availability

The raw data supporting the conclusions of this article will be made available by the authors, without undue reservation.
