# Supplementary material for: Association of Life’s Essential 8 with the prevalence and mortality of chronic obstructive pulmonary disease
Source: Front Med (Lausanne). 2025 Apr 25;12:1530493. doi: 10.3389/fmed.2025.1530493 (PMC12061666; doi:10.3389/fmed.2025.1530493)
Supplement: Supplementary file 1 [file Table_1.docx]

**Supplemental Materials**

**Association of Life’s Essential 8 with All-Cause and Cardiovascular Disease Mortality among individuals with Chronic Obstructive Pulmonary Disease**

Yushan Shi^1#^, Jiafeng Zhang^2#^, Huangrong Song^3#^, Shuangshuang Pu^1^, Chunlai Zhang^1^, Kanghong Xu^1^, Yaobei Liu^1^, Ning Huang^1*^, Feifan Lu^4*^

*Corresponding authors:

Feifan Lu, Department of Gynecology and Obstetrics, Changhai Hospital, Naval Medical University, Shanghai, China. E-mail:luff94@163.com

Ning Huang, Department of Respiratory and Critical Care Medicine, Affiliated Hospital of Shandong University of Traditional Chinese Medicine, Jinan, Shandong, 250000, China. E-mail: amanda678@sina.com

**Table of Contents**

Supplementary Table S12

Supplementary Table S25

Supplementary Table S36

Supplementary Table S47

**Supplementary Table S1. Definition and scoring approach for the American Heart Association’s Life’s Essential 8 score.**

| Domain | CVH Metric | Measurement | Quantification and Scoring of CVH Metric |
| --- | --- | --- | --- |
| Health Behaviors | Diet | Healthy Eating Index-2015 diet score percentile | Quantiles of DASH-style diet adherence  **Scoring (Population):**  Points Quantile  100 ≥95^th^ percentile (top/ideal diet)  80 75^th^ – 94^th^ percentile  50 50^th^ – 74^th^ percentile  25 25^th^ – 49^th^ percentile  0 1^st^ – 24^th^ percentile (bottom/least ideal quartile) |
|  | Physical activity | Self-reported minutes of moderate or vigorous physical activity per week | **Metric:** Minutes of moderate (or greater) intensity activity per week  **Scoring:**  Points Minutes  100 ≥150  90 120 – 149  80 90 – 119  60 60 – 89  40 30 – 59  20 1 – 29  0 0 |
|  | Nicotine exposure | Self-reported use of cigarettes or inhaled nicotine- delivery system | **Metric:** Combustible tobacco use and/or inhaled NDS use; or secondhand smoke exposure  **Scoring:**  Points Status  100 Never smoker  75 Former smoker, quit ≥5 yrs  50 Former smoker, quit 1 - <5 yrs  25 Former smoker, quit <1 year, or currently using inhaled NDS  0 Current smoker  Subtract 20 points (unless score is 0) for living with active indoor smoker in home |
|  | Sleep health | Self-reported average hours of sleep per night | **Metric:** Average hours of sleep per night  **Scoring:**  Points Level  100 7 – <9  90 9 – <10  70 6 – <7  40 5 – <6 or ≥10  20 4 – <5  0 <4 |
| Health Factors | Body mass index | Body weight (kg) divided by height squared (m^2^) | **Metric:** Body mass index (kg/m^2^)  **Scoring:** Points Level 100 <25  70 25.0 – 29.9  30 30.0 – 34.9  15 35.0 – 39.9  0 ≥40.0 |
|  | Blood lipids | Plasma total and HDL-cholesterol with calculation of non-HDL-cholesterol | **Metric:** Non-HDL-cholesterol (mg/dL)  **Scoring:**  Points Level  100 <130  60 130 – 159  40 160 – 189  20 190 – 219  0 ≥220  If drug-treated level, subtract 20 points |
|  | Blood glucose | Fasting blood glucose or casual hemoglobin A1c | **Metric:** Fasting blood glucose (mg/dL) or Hemoglobin A1c (%)  **Scoring:**  Points Level  100 No history of diabetes and FBG <100 (or HbA1c < 5.7)  60 No diabetes and FBG 100 – 125 (or HbA1c 5.7-6.4) (Pre-diabetes)  40 Diabetes with HbA1c <7.0  30 Diabetes with HbA1c 7.0 – 7.9  20 Diabetes with HbA1c 8.0 – 8.9  10 Diabetes with Hb A1c 9.0 – 9.9  0 Diabetes with HbA1c ≥10.0 |
|  | Blood pressure | Appropriately measured systolic and diastolic blood pressure | **Metric:** Systolic and diastolic blood pressure (mm Hg)  **Scoring:**  Points Level  100 <120/<80 (Optimal)  75 120-129/<80 (Elevated)  50 130-139 or 80-89 (Stage I HTN)  25 140-159 or 90-99  0 ≥160 or ≥100  Subtract 20 points if treated level |

**Reference**

1. Lloyd-Jones DM, Allen NB, Anderson CAM, et al. Life's Essential 8: Updating and Enhancing the American Heart Association's Construct of Cardiovascular Health: A Presidential Advisory From the American Heart Association. *Circulation*. Aug 2 2022;146(5):e18-e43.
2. Lloyd-Jones DM, Ning H, Labarthe D, et al. Status of Cardiovascular Health in US Adults and Children Using the American Heart Association's New "Life's Essential 8" Metrics: Prevalence Estimates From the National Health and Nutrition Examination Survey (NHANES), 2013 Through 2018. *Circulation*. Sep 13 2022;146(11):822-835.

**Supplementary Table S2. Healthy Eating Index-2015 Components and Scoring Standards ^a^**

| **Component** | **Maximum points** | **Standard for maximum score** | **Standard for minimum score of zero** |
| --- | --- | --- | --- |
| ***Adequacy ^b^*** | | | |
| Total Fruits | 5 | ≥0.8 cup equiv. per 1,000 kcal | No Fruit |
| Whole Fruits | 5 | ≥0.4 cup equiv. per 1,000 kcal | No Whole Fruit |
| Total Vegetables | 5 | ≥1.1 cup equiv. per 1,000 kcal | No Vegetables |
| Greens and Beans | 5 | ≥0.2 cup equiv. per 1,000 kcal | No Dark Green Vegetables or Legumes |
| Whole Grains | 10 | ≥1.5 oz equiv. per 1,000 kcal | No Whole Grains |
| Dairy | 10 | ≥1.3 cup equiv. per 1,000 kcal | No Dairy |
| Total Protein Foods | 5 | ≥2.5 oz equiv. per 1,000 kcal | No Protein Foods |
| Seafood and Plant Proteins | 5 | ≥0.8 oz equiv. per 1,000 kcal | No Seafood or Plant Proteins |
| Fatty Acids | 10 | (PUFAs + MUFAs)/SFAs ≥2.5 | (PUFAs+MUFAs)/SFAs ≤1.2 |
| **Moderation ^c^** | | | |
| Refined Grains | 10 | ≤1.8 oz equiv. per 1,000 kcal | ≥4.3 oz equiv. per 1,000 kcal |
| Sodium | 10 | ≤1.1 gram per 1,000 kcal | ≥2.0 grams per 1,000 kcal |
| Added Sugars | 10 | ≤6.5% of energy | ≥26% of energy |
| Saturated Fats | 10 | ≤8% of energy | ≥16% of energy |

^a^ Intakes between the minimum and maximum standards are scored proportionately.

^b^ Adequacy components represent the food groups, subgroups, and dietary elements that are encouraged. For these components, higher scores reflect higher intakes, because higher intakes are desirable.

^c^ Moderation components represent the food groups and dietary elements for which there are recommended limits to consumption. For moderation components, higher scores reflect lower intakes, because lower intakes are more desirable.

**Reference**

1. Krebs-Smith SM, Pannucci TE, Subar AF, et al. Update of the Healthy Eating Index: HEI-2015. J Acad Nutr Diet. Sep 2018;118(9):1591-1602.

2. National Cancer Institute. HEI Scoring Algorithm. Accessed August, 2022. https://epi.grants.cancer.gov/hei/hei-scoring-method.html

Supplementary Table S3. Sensitivity analysis for the association of Life’s Essential 8 score with all-cause and cardiovascular mortality by excluding deaths that occurred during the first 2-year follow-up

| Variable | Cases/participants(%) | Model 1 | *P* value |  | Model 2 | *P* value |  | Model 3 | *P* value |
| --- | --- | --- | --- | --- | --- | --- | --- | --- | --- |
|  |  | HR (95%CI) |  |  | HR (95%CI) |  |  | HR (95%CI) |  |
| All-cause mortality |  |  |  |  |  |  |  |  |  |
| Total CVH score | 274 (28.2) | 0.86 (0.79~0.93) | <0.001 |  | 0.79 (0.73~0.87) | <0.001 |  | 0.88 (0.8~0.97) | 0.009 |
| Subgroups |  |  |  |  |  |  |  |  |  |
| Low CVH | 91 (32.4) | 1(Ref) |  |  | 1(Ref) |  |  | 1(Ref) |  |
| Moderate CVH | 173 (27.3) | 0.74 (0.58~0.96) | 0.023 |  | 0.66 (0.51~0.86) | 0.002 |  | 0.81 (0.62~1.07) | 0.136 |
| High CVH | 10 (17.9) | 0.44 (0.23~0.85) | 0.015 |  | 0.27 (0.14~0.53) | <0.001 |  | 0.47 (0.23~0.92) | 0.028 |
| *P* for trend |  |  | 0.003 |  |  | <0.001 |  |  | 0.023 |
| CVD mortality |  |  |  |  |  |  |  |  |  |
| Total CVH score | 49 (5) | 0.83 (0.68~1.01) | 0.057 |  | 0.76 (0.62~0.94) | 0.013 |  | 0.81 (0.65~1.02) | 0.073 |
| Subgroups ^e^ |  |  |  |  |  |  |  |  |  |
| Low CVH | 17 (6) | 1(Ref) |  |  | 1(Ref) |  |  | 1(Ref) |  |
| Moderate CVH | 30 (4.7) | 0.7 (0.39~1.27) | 0.244 |  | 0.62 (0.34~1.14) | 0.122 |  | 0.69 (0.37~1.31) | 0.258 |
| High CVH | 2 (3.6) | 0.5 (0.11~2.15) | 0.348 |  | 0.31 (0.07~1.38) | 0.126 |  | 0.44 (0.1~2.07) | 0.301 |
| P for trend |  |  | 0.182 |  |  | 0.05 |  |  | 0.177 |

CVH, cardiovascular health; CVD, cardiovascular disease; HR, hazard ratio; CI, confidence interval.

Model 1 was not adjusted;

Model 2 was adjusted for age, sex, race/ethnicity, and marital status.

Model 3 was adjusted for age, sex, race/ethnicity, marital status, educational level, PIR, CVD history, CKD history, drinking status, and

depression.

Supplementary Table S4. Sensitivity analysis for the association of Life’s Essential 8 score with all-cause and cardiovascular mortality according to quartile of Life’s Essential 8 score.

| Variable | Model 1 | *P* value |  | Model 2 | *P* value |  | Model 3 | *P* value |
| --- | --- | --- | --- | --- | --- | --- | --- | --- |
|  | HR (95%CI) |  |  | HR (95%CI) |  |  | HR (95%CI) |  |
| All-cause mortality |  |  |  |  |  |  |  |  |
| Q1(<48.0) | 1(Ref) |  |  | 1(Ref) |  |  | 1(Ref) |  |
| Q2(48.0-58.0) | 1.06 (0.8~1.41) | 0.693 |  | 0.94 (0.7~1.25) | 0.672 |  | 1.06 (0.79~1.42) | 0.703 |
| Q3(59-68.5) | 0.76 (0.57~1.02) | 0.072 |  | 0.61 (0.45~0.82) | 0.001 |  | 0.75 (0.55~1.02) | 0.065 |
| Q4(>68.5) | 0.53 (0.38~0.72) | <0.001 |  | 0.46 (0.33~0.64) | <0.001 |  | 0.67 (0.48~0.95) | 0.023 |
| *P* for trend |  | <0.001 |  |  | <0.001 |  |  | 0.005 |
| CVD mortality |  |  |  |  |  |  |  |  |
| Q1(<48.0) | 1(Ref) |  |  | 1(Ref) |  |  | 1(Ref) |  |
| Q2(48.0-58.0) | 0.88 (0.47~1.67) | 0.704 |  | 0.81 (0.42~1.55) | 0.518 |  | 0.88 (0.45~1.71) | 0.707 |
| Q3(59-68.5) | 0.61 (0.31~1.2) | 0.155 |  | 0.49 (0.25~0.97) | 0.042 |  | 0.6 (0.3~1.22) | 0.162 |
| Q4(>68.5) | 0.39 (0.18~0.83) | 0.015 |  | 0.36 (0.16~0.77) | 0.009 |  | 0.48 (0.21~1.09) | 0.08 |
| *P* for trend |  | 0.008 |  |  | 0.003 |  |  | 0.046 |

Abbreviations: HR, Hazard Ratio; CI, Confidence interval; CVD, Cardiovascular disease; Ref, reference.

Model 1 was not adjusted;

Model 2 was adjusted for age, sex, race/ethnicity, and marital status.

Model 3 was adjusted for age, sex, race/ethnicity, marital status, educational level, PIR, CVD history, CKD history, drinking status, and

depression.
